# Medical and Physical Intelligence

**Published:** 1803-09-01

**Authors:** 


### MEDICAL AND PHYSICAL INTELLIGENCE.

*Violent Effects of Oxygenated Muriat of Mercury; communicated to the Medical Repository of New York, by* Dr. JAMES S. STRINGHAM.

I lately had occasion to have recourse to Mr. Addington's manner of curing gonorrhœa, a particular account of which I met with in the sixth volume of the Medical and Chirurgical Review. A married gentleman in this city had (during the absence of his wife) been attacked with symptoms of this disease. A few days afterwards he applied to me for a remedy which would remove it in the shortest time possible, as he was in daily expectation of his wife's return. He added, that should this take place before his complaint was entirely cured, his domestic comfort would be for ever destroyed. Placing some confidence in the assertions of Mr. Addington, I informed him that I did not despair of being able to accomplish his wishes in the course of a week; but that the remedies dies I should employ were such as I had never before used; and that, as they were violent, nothing but the peculiarity of situation could induce me even to give them a trial. On his declaring himself perfectly willing to encounter every hazard, I immediately prepared for him three grains of corrosive sublimate, dissolved in one ounce of rectified spirit of wine. Of this solution he was to take one-half at bed-time, and on the succeeding morning one ounce of Glauber's salts. On the next evening but one he was to repeat the remaining portion, with the salts as formerly. He punctually complied with these directions, and called upon me on the fifth day from the time he had taken the first dose, in no respect better than before. Considering him a proper subject for prosecuting the experiment, and wishing to satisfy myself as to the efficacy of the remedy, I ordered him, two days afterwards, to repeat the solution in the same way as before, taking the salts between each interval. On the night after having swallowed the second dose I was called upon to visit him. He had a profuse salivation, violent retching of the stomach, griping pains in the bowels, with great uneasiness in the head and throat, attended with delirium. He continued in this situation during the whole of the night; the same remedies were prescribed as though he had been poisoned by corrosive sublimate. On the next day the violence of his symptoms was considerably mitigated, and in about twenty-four hours afterwards they entirely disappeared. His gonorrhœa, however, continued precisely the same, and, in three weeks afterwards, was cured by the plan generally adopted.

From this single trial, I have no hesitation to conclude, that the method of curing gonorrhœa, as proposed by Mr. Addington, is both dangerous and uncertain. The same objections will not apply here that were urged against the surgeon who denied the efficacy of this mode of treatment. The directions of Mr. Addington were in this case literally pursued, so far as common humanity would permit; but without producing the smallest benefit to the patient.

I wish to make this case public through the medium of your useful work, with the hope that it may be the mean of preventing any other practitioner being placed in the same unpleasant dilemma from which I have been so lately extricated.

---

*Curious Analogy between the Motion of the Blood in the Arteries, and of Water in Troughs and Conduits; in a Letter from* Moses Younglove, *Esq. to* Dr. Mitchell, *dated New-Lebanon Springs, January* 15, 1803.

The ingenious and profound Dr. E. Darwin, in his Zoonomia, vol. i. § 12, 1 et seq. and elsewhere in his work, very rationally, and perhaps demonstratively, accounts for the systole and diastole of the heart and arteries, as well as for the alternation of muscular exertion and quiescence in other parts, by the mere principle of *animation;* but it is a curious fact, that, from some physical or chemical chemical cause beyond my comprehension, something similar to pulsation occurs in the running of water in an aqueduct. I have observed it in several, but most frequently and particularly in the one which accommodates my kitchen, barn-yard and hog-pen. There is an uninterrupted descent of perhaps twelve degrees below the level of the horizon in the whole distance from the fountain to my house (66 rods), in which the rapid current is only broken by a watering-post, which, in a dry time, waters several fields bordering on it. As the water ascends in a bored channel on one side of the post, to the height of seven or eight feet, and descends in a like channel on its other side, I at first imagined it might be partly or wholly owing to this ascent the flow of water in the duct is sometimes very small, slow and still, and then swift and full, with a roaring noise, and that in regular alternations of about five minutes; but I have since found that the same regular vicissitudes of flowing occur above as well as below the post.

The fountain does not afford a sufficient stream to fill the duct except in great rains; but when it runs fullest, and when it runs most scantily, the same regular succession in flowing continues.

Suppose, then, that I could construct my aqueduct anew, of some substance as flexible and elastic as a human artery, bored smaller, to fit the ordinary stream from my fountain, would not these regular alternations of flowing in it occasion a dilatation and contraction of it somewhat similar to arterial pulsation?

The above phenomenon I do not remember ever to have heard or read of; and, trivial as it seems, it may, perhaps, form a link in some important chain of research. If you think it merits publication, it is offered to the service of yourself and the public.

---

The Winter Courses of Lectures given at the adjoining Hospitals of St. Thomas's and Guy's, will commence in the following order: At St. Thomas's Hospital, Anatomy, and the Operations of Surgery, by Mr. CLINE and Mr. ASTLEY COOPER, on Saturday, October 1, at one o'clock.—At Guy's Hospital, Practice of Medicine, by Dr. BABINGTON and Dr. CURRY, on Monday, October 3, at ten in the morning.—Principles and Practice of Chemistry, by Dr. BABINGTON and Mr. ALLEN, on Tuesday, October 4, at ten in the morning.—Theory of Medicine and Materia Medica, by Dr. CURRY, on Tuesday, October 4, at seven in the evening.—Midwifery and Diseases of Women and Children, by Dr. HAIGHTON, on Wednesday, October 5, at eight in the morning.—Physiology, or Laws of the Animal Economy, by Dr. HAIGHTON, on Monday, October 10, at a quarter before seven in the evening.—Principles and Practice of Surgery, by Mr. ASTLEY COOPER, illustrated by select cases under his care in Guy's Hospital, on Monday, October 10, at eight in the evening. The above lectures given at these two hospitals, are so arranged, that no two of them interfere with each other in the hours of attendance, and the whole s calculated to form a *complete course of medical and chirurgical instruction.* The terms and other particulars to be learned by applying to Mr. Stocker, apothecary to Guy's Hospital; who is also empowered to enter gentlemen as Pupils to such Lectures as are delivered at Guy's.

The Autumnal Course of Lectures will begin at the Medical Theatre, St. Bartholomew's Hospital, in the following order:

On the Theory and Practice of Medicine, by Dr. ROBERTS and Dr. POWELL.—Clinical Lectures on select Cases occurring in the Hospital, will be occasionally given by Dr. ROBERTS.—On Anatomy and Physiology, by Mr. ABERNETHY.—On Comparative Anatomy and Physiology, by Mr. MACARTNEY.—On the Theory and Practice of Surgery, by Mr. ABERNETHY.—On Chemistry and the Materia Medica, by Dr. POWELL.—On Midwifery and the Diseases of Women and Children, by Dr. THYNNE.—The Anatomical Lectures will begin on Saturday, Oct. 1, at two o'clock, and the other Lectures will begin in the following week. Further Particulars may be known by applying to Mr. Nicholson, at the apothecary's shop, St. Bartholomew's Hospital.

THEATRE, LONDON HOSPITAL. On Saturday, October 1, at two o'clock, Mr. HEADINGTON and Mr. FRAMPTON will commence their Lectures on Anatomy, Physiology, and the Principles and Operations of Surgery. Demonstrations as usual by Mr. ARMIGER.

The Lectures of Dr. PEARSON will commence in Leicester-square, the second week in October next. A lecture is given on Therapeutics from a quarter before to a quarter past eight o'clock; on the Practice of Physic, from a quarter past eight to about nine o'clock; and on Chemistry, from a quarter after nine to ten o'clock, every morning except Saturdays, on which days a Lecture is delivered on the Practice of Physic from eight to nine, and on the Cases of Patients from nine to ten o'clock.

Dr. BRADLEY will commence his Course of Lectures on the Theory and Practice of Medicine, in the first Week in October. Particulars may be had at his house, No. 25, Parliament Street.

Dr. BATTY's Course of Lectures on the Theory and Practice of Midwifery, and on the Diseases of Women and Children, will commence on Monday, October 10, at his house in Great Marlborough Street.

Dr. DENNISON and Dr. SQUIRE, on Saturday, October 1, will begin a Course of Lectures on the Theory and Practice of Midwifery, and the Diseases of Women and Children.

Dr. CLARKE will begin a Course of his Lectures on the Practice of Midwifery and the Diseases of Women and Children, on Tuesday, October 4, at a quarter past ten o'clock. These Lectures are given in the winter season, only, at the house of Dr. Clarke, No. 1, New Burlington Street.

Mr.

Mr. Blair's Physiological Lectures, will commence at the Bloomsbury Dispensary, Great Russel Street, on Tuesday evening, October 4, at eight o'clock precisely.

A printed Syllabus, price five shillings, may be had at the Lecture Room.

Mr. Wilson and Mr. Thomas, of the Theatre of Anatomy, Great Windmill Street, will begin their Winter Course of Lectures on Anatomy, Physiology, Pathology, and Surgery, on Monday, October 3, at two o'clock. Practical Anatomy will be continued as usual. A plan of the Course may be had by applying to Mr. Wilson, in Great Windmill Street, or to Mr. Thomas, in Leicester Square.

Mr. Taunton will commence his Lectures on Anatomy, Physiology, and Surgery, in the beginning of October. The general objects of them are to explain the structure and functions of the human body; to shew the situation and connection of the different organs with each other; and to take some notice of their transitions from health to disease, which will be demonstrated by preparations of the parts, drawings, and recent dissections, for the information of medical students, professional men, and scientific persons; and will be given nearly in the following order: Some general Lectures, explaining the structure and economy of the component parts of an animal body; then, Osteology, or the skeleton, will be explained, and the separate bones described, their different modes of union, including ligaments, joints, and luxations. Thoracic viscera, including the heart and lungs, with the laws of circulation and respiration. Abdominal viscera, stomach, alimentary canal, and digestion. Organs of generation, male and female. Arteries, veins, and lymphatics. Brain and nerves. The organs of the different senses, the ear, eye, nose, mouth, and common integuments. The art of making anatomical preparations. Particulars may be known by applying to Mr. Bartlet, Finsbury Dispensary, St. John's Square, Clerkenwell.

Mr. Chevalier, Surgeon of the Westminster General Dispensary, will commence his next Course of Lectures on the Principles and Operations of Surgery, on Wednesday the 5th of October, at seven o'clock in the evening, at his house in South Audley Street, Grosvenor Square, where a printed Prospectus may be had.

Mr. Carpue, Surgeon to the Forces, will commence his Lectures on Anatomy and Surgery, at his Theatre, Broad Street, Golden Square, on Monday, September 26. Mr. C's plan prevents him from taking more than ten pupils each course. The dissecting room will be open from seven o'clock in the morning till the evening. Particulars may be known by applying to Mr. Carpue, Leicester Square.

